# The chemo-gut study: investigating the long-term effects of chemotherapy on gut microbiota, metabolic, immune, psychological and cognitive parameters in young adult Cancer survivors; study protocol

**DOI:** 10.1186/s12885-019-6473-8

**Published:** 2019-12-23

**Authors:** Julie M. Deleemans, Faye Chleilat, Raylene A. Reimer, Jan-Willem Henning, Mohamad Baydoun, Katherine-Ann Piedalue, Andrew McLennan, Linda E. Carlson

**Affiliations:** 1 Cumming School of Medicine, Division of Medical Science, University of Calgary, Calgary, Canada; 2 Cumming School of Medicine, Division of Psychosocial Oncology, University of Calgary, Calgary, Canada; 3 Faculty of Kinesiology, University of Calgary, Calgary, Canada; 4 Department of Biochemistry and Molecular Biology, Cumming School of Medicine, Calgary, Canada; 5 Department of Oncology, Tom Baker Cancer Centre, Calgary, Canada

**Keywords:** Young adult, Cancer, Chemotherapy, Gut microbiota, Psycho-oncology, Cytokines, Cortisol

## Abstract

**Background:**

The gut microbiota is an important modulator of immune, metabolic, psychological and cognitive mechanisms. Chemotherapy adversely affects the gut microbiota, inducing acute dysbiosis, and alters physiological and psychological function. Cancer among young adults has risen 38% in recent decades. Understanding chemotherapy’s long-term effects on gut microbiota and psycho-physiological function is critical to improve survivors’ physical and mental health, but remains unexamined. Restoration of the gut microbiota via targeted therapies (e.g. probiotics) could potentially prevent or reverse the psycho-physiological deficits often found in young survivors following chemotherapy, ultimately leading to reduced symptom burden and improved health.

**Methods:**

This longitudinal study investigates chemotherapy induced long-term gut dysbiosis, and associations between gut microbiota, and immune, metabolic, cognitive and psychological parameters using data collected at < 2 month (T1), 3–4 months (T2), and 5–6 months (T3) post-chemotherapy. Participants will be 18–39 year old blood or solid tumor cancer survivors (*n* = 50), and a healthy sibling, partner or friend as a control (*n* = 50). Gut microbiota composition will be measured from fecal samples using 16 s RNA sequencing. Psychological and cognitive patient reported outcome measures will include depression, anxiety, post-traumatic stress disorder symptoms, pain, fatigue, and social and cognitive function. Dual-energy X-ray Absorptiometry (DXA) will be used to measure fat and lean mass, and bone mineral concentration. Pro-inflammatory cytokines, C-reactive protein (CRP), lipopolysaccharide (LPS), serotonin, and brain derived neurotrophic factor (BDNF) will be measured in serum, and long-term cortisol will be assayed from hair. Regression and linear mixed model (LMM) analyses will examine associations across time points (T1 – T3), between groups, and covariates with gut microbiota, cognitive, psychological, and physiological parameters.

**Conclusion:**

Knowing what bacterial species are depleted after chemotherapy, how long these effects last, and the physiological mechanisms that may drive psychological and cognitive issues among survivors will allow for targeted, integrative interventions to be developed, helping to prevent or reverse some of the late-effects of treatment that many young cancer survivors face. This protocol has been approved by the Health Research Ethics Board of Alberta Cancer Committee (ID: HREBA.CC-19-0018).

## Background

The digestive tract is the most densely colonized microbial ecosystem in the body. The bacteria that make up the majority of this ecosystem (in addition to fungi, viruses and archaea) are important for immune, metabolic, psychological and cognitive function. A disruption in the microbiota, termed dysbiosis may induce aberrant neurophysiological function and behaviour [1], such as increased anxiety and depressive behaviour [2] as well as impaired immune function characterized by increased systemic inflammation in humans and rodent models [3, 4]. Dysbiosis-induced systemic inflammation can independently and bidirectionally activate or exacerbate stress circuits, most notably the hypothalamic-pituitary-adrenal (HPA)-axis, via the vagus nerve [2, 5].Cancer treatments, especially chemotherapy, can drive microbial dysbiosis in the gut [6], alter immune, metabolic and HPA-axis function [7], as well as cause psychological and cognitive perturbations both acutely and, potentially in the longer-term [8, 9].

**Fig. 1:**
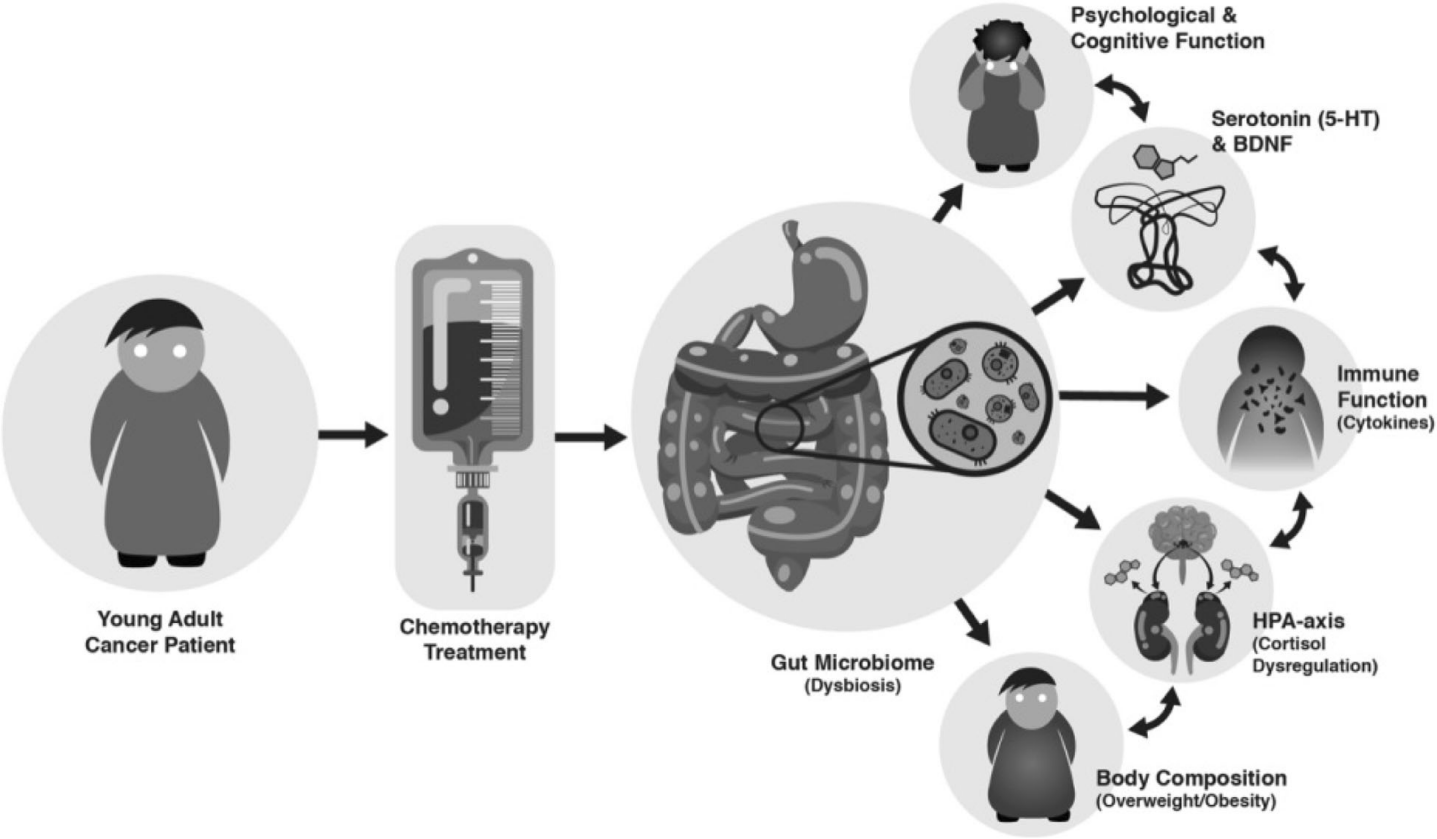
Chemotherapy Driven Dysbiosis of the Microbiota-Gut-Brain Axis. The Chemotherapy Driven Dysbiosis of the Microbiota-Gut-Brain Axis model posits that chemotherapy given to young cancer patients induces long-term gut dysbiosis, increasing intestinal permeability (i.e. “leaky gut”), which allows bacterial toxins, such as lipopolysaccharide (LPS), to enter the blood stream. This subsequently leads to systemic inflammation via increases in pro-inflammatory cytokines, especially IL-6, IL-1b, and TNF-a, and C-reactive protein, as well as dysregulation of the HPA-axis. This creates a feedback loop in which inflammatory mechanisms trigger the stress response to increase systemic cortisol, feeding back into the immune system to exacerbate levels of inflammation. Gut microbiota dysbiosis and dysregulation of the immune system and HPA-axis attenuate levels of serotonin (5-HT) and brain derived neurotrophic factor (BDNF), which then work in tandem to induce changes in psychological and cognitive function, including increased symptoms of anxiety and depression (i.e. “sickness behaviours”), pain, fatigue, and social and cognitive deficits. Finally, gut dysbiosis, immune, and HPA-axis dysregulation may also augment patients’ vulnerability to increased adipose tissue, and subsequent overweight and obesity, further compromising their health

The incidence of cancer among young adults has risen nearly 40% in recent decades [10]. Early adulthood is an important period for psychological and cognitive development, which is influenced by biological and environmental factors, including the gut microbiota [11]. Understanding chemotherapy’s long-term effects on the gut microbiota and subsequent relationships with immune, metabolic, psychological and cognitive outcomes is of critical importance in order to improve young adult cancer survivors’ physical and psychosocial health, but remains unexamined. Therefore, based on the following model (Fig. 1), we plan to investigate whether chemotherapy treatments are related to long-term gut microbiota dysbiosis, and subsequent associations with immune, metabolic, psychological and cognitive outcomes.

### Cancer in young adults

The incidence of cancer among young adults has dramatically increased in recent decades [10], with over 8000 young Canadians diagnosed annually [12]. Between 1992 and 2013 the incidence of cancer has increased among young Canadian’s aged 15 to 29 by 18.2, and 11.9% among 30 to 39 year olds [12]. Considering their age at diagnosis, young adults experience unique personal, physical, and socioeconomic challenges throughout their cancer experience [12, 13]. As most young adult survivors are exposed to toxic therapies (e.g. chemotherapy) many will face years of chronicity living with treatment-related physical and psychosocial side-effects. For instance, Asher [14] reports that executive functioning, attention, concentration, processing speed, reaction time, motor speed and dexterity were the cognitive domains most frequently associated with chemotherapy induced cognitive impairment in all cancer populations. Additionally, Seitz et al. [15] examined the prevalence of posttraumatic stress, depression and anxiety among young adult survivors (*n* = 820) of adolescent cancer with a mean current age of 30.4 years [15]. They found that 90.5% cancer survivors had received chemotherapy, and 22.4% reported clinical symptoms of posttraumatic stress, anxiety and/or depression compared to only 14.0% of controls [15]. Posttraumatic stress symptoms in both male and female survivors were found to be more than three times that of controls [15]. Furthermore, female survivors reported symptoms of depression and anxiety significantly more often than controls, and 24.3% of the survivors met DSM-IV criteria for at least one mental disorder, relative to only 15.3% of the controls [15]. Indeed, the increased prevalence of young adult cancers in conjunction with the improved survival rates has resulted in a large group of survivors facing years of chronicity, unique challenges, and specific needs emerging from their cancer experience.

### Chemotherapy and gut microbiota

While the detrimental effects of chemotherapy on psychosocial and cognitive function are established, evidence for the underlying physiological mechanisms and system interactions that drive the psychological and cognitive impairments remains limited. The gut-brain axis refers to a bidirectional communication system within an organism that enables gut microbes to communicate with the brain and vice versa via neural, endocrine, immune and metabolic pathways [4, 16]. The role of the gut-brain axis in the context of the young adult is a critical consideration, since environmental toxins can interfere with normal development, potentially increasing individuals’ risk for developing psychiatric disorders such as depression and anxiety, which typically develop during adolescence and young adulthood.

Chemotherapy has been shown to adversely affect the gut microbiota in both pediatric and adult cancer cohorts. For example, Huang et al. [17] investigated changes in gut microbiota among 36 children with acute lymphoblastic leukemia (ALL) before and after receiving high-dose methotrexate chemotherapy, finding that the total gut microbial content in fecal samples decreased by 29.6%, relative to healthy controls, with significant reductions in Bifidobacteria*, Lactobacillus* and *E. coli* [17]. Furthermore, using a pre-post-measure design, Montassier et al. [18] examined 28 adult non-Hodgkin’s lymphoma patients treated with chemotherapy to assess the functional mechanisms of the gut microbiota in mediating the pathophysiology of mucositis. Mucositis is a condition characterized by painful inflammation and ulceration of the mucous membranes lining the digestive tract, affecting 70% of patients following chemotherapy [18, 19]. Significant decreases in Firmicutes and Actinobacteria, and increases in the abundance of Proteobacteria were observed alongside a reduced capacity for nucleotide, energy and vitamin metabolism, suggesting that chemotherapy induces severe compositional and functional imbalance in the gut microbiome that is associated with gastrointestinal mucositis [18]. While recent research demonstrates that chemotherapy is acutely associated with dysbiosis, research has not yet examined whether these effects persist in the longer-term (i.e. over months) following the cessation of chemotherapy. Therefore, as part of this study we will conduct a novel examination of the longer-term effects of chemotherapy on gut microbiota up to 6-months post-chemotherapy.

### Gut microbiota, immune function, the HPA-axis and chemotherapy

The gut-brain axis exerts regulatory effects on immune function and behaviour. Indeed, previous research has found that under certain conditions the protective epithelial layer in the gut can become compromised leading to increased intestinal permeability and consequently increased systemic circulation of the bacterial endotoxin, lipopolysaccharide (LPS), a component of the cell wall of Gram-negative bacteria [11, 20]. This “leaky gut” results in a peripheral inflammatory immune response, such that systemic levels of pro-inflammatory cytokines are increased [20], inducing sickness behaviours [21].

The potential for gut microbiota to affect the immune system and inflammatory mechanisms is a critical consideration in the context of anxiety and depression, as inflammation has been implicated as a driving factor in depressive illness [3, 22]. Behaviours indicative of sickness include lethargy, depression, anxiety, anorexia, social withdrawal and isolation, and have consistently been associated with augmented levels of pro-inflammatory cytokines, especially tumour necrosis factor alpha (TNF-α), interleukin 6 (IL-6), and interleukin 1 beta (IL-1ß) [21]. Additionally, studies involving Interferon alpha (INF-α) treatment for melanoma patients often report the development of cytokine induced depressive symptoms and sickness behaviours following INF-α treatment [23].

The HPA-axis may also play a role in mediating gut-brain axis functions. Previous studies have shown that gut bacteria can activate stress circuits via the vagus nerve [2], which modulates gut-brain axis function, subsequently affecting behaviour [5]. Corticotrophin releasing factor (CRF), a stress-related neuropeptide, is highly associated with gut-brain axis functioning [24]. Furthermore, results from human and animal studies have found that increased systemic inflammation (e.g. augmented pro-inflammatory cytokines from endotoxemia) is associated with increased levels of adrenocorticotropic hormone (ACTH) and cortisol, further implicating the role of immune function in moderating HPA-axis function [25]. As evidence continues to accumulate demonstrating that the gut-brain axis contributes to physiological function and affective behaviour related to the HPA-axis and immune system, it is important to consider the potential bi-directional communication between these systems, particularly in the context of disease.

Chemotherapy has been associated with increased intestinal wall permeability, mucositis, and the onset of a systemic inflammatory immune response, dysregulation of the HPA-axis, and subsequent manifestation of sickness behaviours [11, 20, 21]. Gut microbiota may play a role in mediating such effects [7]. Moreover, in addition to adversely affecting the gut microbiota, chemotherapy-induced mucositis is also associated with significant elevations in systemic pro-inflammatory cytokines, especially IL-1ß and IL-6 [26], which are accompanied by increased symptoms of pain, anxiety, and depression [27]. As such, the potential disruption of normal physiological and psychological development among young cancer survivors and the role of gut microbiota in moderating such changes is of particular concern, as many survivors face lifelong health challenges following a cancer experience, which is why these immune and metabolic components have been included within our study.

### Gut microbiota, and psychological and cognitive function

Gut microbiota dysbiosis has been implicated in cognitive and psychological deficits. Previous studies demonstrate that alterations in gut microbiota can affect both anxiety and depressive behaviour in animals and humans [2, 28–31]. Neurotransmitter dysregulation is widely implicated in depressive illness, and certain neurotransmitters including serotonin (5-HT), dopamine, GABA (gamma-Aminobutyric acid), and noradrenalin, are affected by the gut microbiota [4, 32]. This is an important consideration given that neurotransmitters, especially serotonin as it participates in mood regulation, are implicated in the neurological dysregulation observed in depressed patients [33] and cancer survivors frequently experience comorbid depression following a cancer experience [15]. Furthermore, a small but growing body of pre-clinical evidence suggests that the gut-brain axis also plays a role in cognitive function, including spatial learning and working memory [34], and alterations in brain derived neurotrophic factor (BDNF) expression in regions of the brain that are associated with specific cognitive functions (e.g. the hippocampus, which is involved in learning and memory) [34, 35].

While the majority of evidence stems from animal research, some human studies have also shown associations between the gut microbiota, and psychological and cognitive function**.** For instance, Prehn-Kristensen et al. [36] investigated the gut microbiota composition of 14 male attention deficit hyperactivity disorder (ADHD) patients compared with healthy male controls, and found that alpha diversity of ADHD patients was significantly decreased, and augmented levels of *Bacteroidaceae*, and both *Neisseriaceae* and *Neisseria spec*. Were identified as potential biomarkers for the ontogeny of juvenile ADHD [36]. Additionally, Naseribafrouei et al. [37] examined associations between human fecal microbiota and depression in 55 adults (37 patients versus 18 controls), finding that Bacteroidales were overrepresented, while *Lachnospiraceae* species were underrepresented among depressed patients. Additionally, the *Oscillibacter* genus, of which valeric acid is the main metabolic end product and a homolog of the neurotransmitter GABA, a neurotransmitter implicated in major depressive disorder, was also correlated with depression [37].

Furthermore, in a triple-blind, placebo-controlled, randomized, pre- and post-intervention study with 40 healthy participants, Steenbergen et al. [29] investigated the effects of a multispecies probiotic versus a placebo control on cognitive reactivity to sad mood. Participants who received the 4-week probiotic intervention showed a significant attenuation in overall cognitive reactivity to sad mood, which appeared to be a function of reduced rumination and aggressive thoughts [29]. Finally, Hilimire et al. [28] conducted a cross-sectional study with 710 young adults to examine whether consumption of fermented foods, which contain probiotics, interacts with neuroticism to predict social anxiety symptoms, using self-reported measures of fermented food consumption, neuroticism, and social anxiety [28]. For individuals reporting high traits of neuroticism, greater consumption of fermented food was associated with fewer symptoms of social anxiety, suggesting that fermented foods containing beneficial probiotic bacteria may exert protective effects against social anxiety symptoms for young adults with higher trait neuroticism [28]. Although the underlying mechanisms mediating these cognitive-behavioural effects remain to be elucidated, it is plausible that alterations in gut microbiota resulting from dietary supplementation with health promoting bacteria play a role.

Chemotherapy has been associated with cognitive and psychological impairments [14, 15]. For example, Prasad et al. [38] examined associations between neurocognitive dysfunction and psychosocial outcomes on the achievement of social milestones among adult survivors of adolescent and early young adult (AeYA) cancer who had been diagnosed between 11 to 21 years of age. Relative to healthy siblings, AeYA survivors’ were more likely to experience neurocognitive dysfunction in domains related to task efficiency, emotion regulation, and memory, were less likely to be married or employed, and reported greater levels of emotional distress characterized by anxiety, somatization, and depression [38]. Given this, the potential role of gut microbiota in mediating or moderating chemotherapy’s effects is an important direction for novel investigation. Such knowledge will help us to better understand the ontogeny of cognitive and psychological deficits in young cancer survivors, while also presenting new opportunities for targeted interventions perhaps via prebiotic and/or probiotic supplementation of key health promoting bacteria that may be depleted following chemotherapy.

### Cancer, gut microbiota and obesity

Findings regarding associations between gut microbiota and obesity also hold implications related to body composition among young adult cancer survivors who tend to be more overweight and obese relative to healthy peers. For example, recent data from a sample of 49 young adult survivors of adolescent cancer found that over 57% of these survivors were overweight or obese, which is higher than the national average [39], and consistent with previous research on obesity in adolescent and young adult (AYA) cancer survivors [40]. In human and animal studies, diet and/or antibiotic induced gut microbiota dysbiosis has been shown to play a strong role in the development of obesity [41–44]. Given the established link between obesity and gut microbiota dysbiosis [45], it is possible that chemotherapy may adversely affect metabolic processes mediated by the gut microbiota, resulting in an increased risk for young adult cancer survivors to become overweight or obese. However, additional research is needed to examine the potential relationship between chemotherapy, gut microbiota, and fat mass among young cancer survivors; a rationale for our inclusion of these elements in our study.

### Present study

#### The objectives of the present study are to investigate

i. Whether chemotherapy is related to long-term attenuation of gut microbiota alpha diversity (i.e. gut dysbiosis, and which species differ significantly between patients and healthy controls);
ii. Associations between gut microbiota, body composition (i.e. fat mass and lean mass, body fat percentage, and bone mineral concentration), inflammatory cytokine biomarkers (IL-6, IL-1B, TNF-a, IL-10), C-reactive protein (CRP), lipopolysaccharide (LPS), serotonin (5-HT), brain derived neurotrophic factor (BDNF), and cortisol;
iii. Associations between gut microbiota, and psychological and cognitive outcomes including anxiety, depression, PTSD symptoms, pain, fatigue, and social and cognitive functioning;
iv. Relationships between demographic and clinical factors, gut microbiota, and physiological, psychological and cognitive outcomes for survivors.

We hypothesize that:

i. Chemotherapy will be associated with significant reductions in gut microbiota alpha diversity at all time points, and relative to healthy controls;
ii. Gut dysbiosis will be associated with increased risk for overweight/obesity, elevated pro-inflammatory cytokines, CRP and LPS, reduced serotonin and BDNF, and augmented levels of cortisol in survivors;
iii. Gut dysbiosis will be associated with increased anxiety, depression, PTSD symptoms, pain, fatigue, and social and cognitive deficits in survivors;
iv. There will be relationships between certain clinical factors (e.g. mucositis, antibiotic use), gut microbiota alpha diversity, and physiological, psychological and cognitive outcomes.

## Methods and design

### Participants

#### Inclusion criteria for survivors

1. Currently between 18 to 39 years of age
2. Diagnosed with a blood cancer or solid tumor
3. Received chemotherapy
4. Currently within 6 months of final chemotherapy treatment
5. Stages I – IV including metastatic, IF patient is off chemotherapy treatment and stable
6. English speaking
7. Access to an electronic device (i.e. computer, tablet, smart phone) with in internet connection

#### Exclusion criteria for survivors

1. CNS or colorectal cancer tumor diagnosis
2. Recipient of an allogenic stem cell transplant
3. Women who are pregnant
4. Having taken antibiotics within 1 month prior to fecal collection
5. Diagnosis of a significant cognitive or developmental delay that precedes cancer diagnosis (e.g. down syndrome, ASD)

#### Inclusion criteria for control participants

1. Currently between the ages of 18 to 39
2. Be a sibling, individual cohabiting with the patient (e.g. roommate, partner), or close friend
3. English speaking
4. Access to an electronic device (i.e. computer, tablet, smart phone) with in internet connection

#### Exclusion criteria for control participants

1. Having taken antibiotics within 3 months prior to fecal collection
2. Personal history of cancer
3. Current diagnosis/medication treatment for anxiety and/or depression
4. Diagnosis of irritable bowel syndrome/disease
5. Women who are pregnant
6. Diagnosis of a significant cognitive or developmental condition (e.g. down syndrome, ASD)

##### Sample size and power

Target sample sizes of at least *n* = 50 cancer survivors and *n* = 50 healthy controls were selected based on sample sizes from previous studies on gut microbiota in cancer patients [17, 18] and practical considerations given the timeline and availability participants. With this sample size (*n* = 100 total), a medium effect size for group differences (Cohen’s d = 0.5, estimated with a power of 0.80, alpha = 0.05) can be detected using a one-sided test of the primary hypothesis that cancer survivors will have lower alpha diversity relative to controls [46].

##### Recruitment

Participants will be recruited in Calgary, Alberta from the Tom Baker Cancer Center, via social media and local advocacy groups, and by mail-out through the Alberta Cancer Registry. Patient referral and consent to contact forms will be provided to oncologists. Recruitment posters will be posted at relevant sites, and a digital copy of the poster will be shared on social media platforms.

### Demographics and clinical data

Clinical and demographic factors including current age, age at diagnosis, income, occupation, ethnicity, education, living arrangements, cancer diagnosis and treatments received, history of treatment related mucositis, current diet and exercise regimes, bowel habits, glucocorticoid medication, antibiotic, and probiotic use within the last 2 years, smoking, birth delivery mode (i.e. caesarian section vs. vaginal), and breastfeeding during infancy will be collected as these variables are known to impact the gut microbiota.

### Gut microbiota profiling

Investigators will provide participants with a stool collection kit and instructions for collecting samples. Stool samples will be collected into a sterile conical tube and placed in a biohazard bag that will be stored in the participant’s freezer until the date of their appointment (up to 3 days). Samples will be brought to the University of Calgary on ice and stored at − 80 °C until processed. Microbial profiling will be carried out according to our previously published protocol [42]. Bacterial DNA will be extracted from stool samples using a FastDNA spin kit for feces (MP Biomedicals, Santa Anna, CA, USA) and the V3-V4 region sequenced by the Centre for Health Genomics and Informatics (University of Calgary) using the Illumina 16S rRNA sequencing platform. Sequencing data from MiSeq will be demultiplexed and converted to fastq format using Illumina’s bcl2fastq software. Primers will be removed from reads using CutAdapt and initial quality trimming performed. Following primer removal, length sequences of shorter than 10 base pairs will be removed, and the quality trimming threshold set at Q20. R package DADA2 will be used for sequence/OTU clustering. Chimeric sequences will then be removed, and taxonomic assignment performed using the RDP classifier and RDP database as reference. Further downstream analysis will be performed using Phyloseq R package. The Shannon and Simpson index will be used to measure alpha diversity. Non-metric multidimensional scaling (NMDS) on Bray-Curtis dissimilarity matrix will be used to evaluate beta diversity. Differential abundance analysis between groups will be carried out using the LEfSe algorithm [47], with an alpha = 0.05 to determine significance of differentially abundant features.

### Metabolic and anthropomorphic outcomes

*Body composition* will be measured using dual-energy-x-ray absorptiometry (DXA) [48]. Specifically, fat mass, lean mass, body fat percentage, and bone mineral concentration will be quantified using whole-body dual-energy x-ray absorptiometry (DXA; Hologic QDR 4500; Hologic, Inc., Bedford, MA), which is a valid and reliable measure of body composition in healthy adolescents and adults, as well as cancer patients [48, 49].

### Biomarker outcomes

*Pro-inflammatory cytokines* interleukin 6 (IL-6), interleukin 1 beta (IL-1ß), and tumour necrosis factor alpha (TNF-α), anti-inflammatory cytokine interleukin 10 (IL-10), and C-reactive protein (CRP) will be measured in serum using MesoScale multiplexing [50], in consideration of the robust plate to plate reproducibility over time and the dynamic range with a lower limit of detection in the sub picograms per milliliter (i.e. pg/mL) range.

*BDNF* will be assayed from blood serum using MesoScale multiplexing [50], in consideration of the robust plate to plate reproducibility over time and the dynamic range with a lower limit of detection in the sub picograms per milliliter (i.e. pg/mL) range.

*Serotonin* will be measured using Serotonin ELISA (enzyme-linked immunosorbent assay) kits from Enzo Life Sciences [51] according to manufactures instructions. The ELISA is a plate-based assay technique designed for detecting and quantifying substances, including neurotransmitters and proteins.

*Lipopolysacchide* (LPS), a gram-negative bacterial endotoxin associated with intestinal permeability, systemic inflammation, and sickness behaviours, will be measured from serum using MesoScale multiplexing [50].

*Cortisol* will be analyzed using hair samples, which provide a reliable and valid measure of long-term cortisol activity [52, 53]. Hair cortisol will be quantified using liquid chromatography tandem mass spectrometry, and is reported to be more representative of total cortisol production relative to single saliva or serum measures, allowing us to quantify cortisol production over an extended period of time.

### Psychological and cognitive measurement

Post-Traumatic Stress symptoms will be measured using the Impact of Events Scale. Depression, anxiety, pain behaviour, fatigue, cognitive function, and social isolation will be measured via the NIH Patient-Reported Outcomes Measurement Information System (PROMIS). PROMIS is a set of person-centered measures that evaluates physical, mental, and social health, and is a valid measure of psychosocial outcomes among young adult cancer patients [54]. Details of these measures can be found in Table 1. An objective measure of executive function will also be collected using the Sustained Attention to Response Task (SART) is a computer-based go/no-go task that is designed to measure working memory, sustained attention, and impulse/inhibitory control [55], cognitive deficits that are frequently experienced by survivors who have received chemotherapy. The SART evaluates participants’ ability to withhold behavioral response to a single, infrequent target presented against a background of frequent non-targets [55]. Participants are asked to respond by pressing a button or the spacebar key after seeing the non-target on the screen and to inhibit their response to seeing the target. This task requires participants to be attentive to their responses, such that, at the appearance of a target, they can override the dominant motor response and substitute the directly antagonistic response (i.e. withhold button press). This study will use the online version of the SART, delivered through the Inquisit web software provider Millisecond.com. In this version of the task, a practice block (~ 30 s) is followed by two blocks (~ 3 min each), totaling approximately 6 min to complete the SART task.

**Table 1:** Psychological and Cognitive Measures

Outcome	Measure	Scoring	Statement Example
Depression	PROMIS - Ca Item Bank v1.0 - Emotional Distress - Depression questionnaire: 30 items assessing presence and severity of depression	Patient rated on 5 point scale from “never” (1) to “always” (5)	“In the past 7 days… I felt helpless”
Anxiety	PROMIS - Ca Item Bank v1.0 - Emotional Distress - Anxiety questionnaire: 22 items re: symptoms of anxiety	Patient rated 5 point scale from “never” (1) to “always” (5)	“In the past 7 days… my worries overwhelmed me”
Post-Traumatic Stress Disorder (PTSD) symptoms	Impact of Life Events Scale: 22 items divided into 3 subscales that include avoidance, intrusion, and hyper-arousal. Evaluates symptom presence, severity, and degree of impairment	Patient rated 5 point scale from “not at all” (0) to “extremely” (4)	“How distressing has each difficulty been for you during the past 7 days with respect to cancer… I had trouble staying asleep”
Social Isolation	PROMIS Item Bank v2.0 - Social Isolation 14 item Short Form: evaluates feelings about perceived social isolation	Patient rated on a 5 point scale from “never” (1) to “always” (5)	“I feel isolated from others”
Pain Behaviour	PROMIS: Item Bank v.2.0 – Pain Behavior scale: 20 items evaluating behaviours related to pain	Patient rated from “Had no pain” (x), Never (1) to “Almost Always” (5)	“In the past 7 days, when I was in pain… I took medication for the pain”
Fatigue	PROMIS: Item Bank v1.0 - Fatigue - Short Form 8a scale: 8 items re: fatigue frequency and intensity	Patient rated on a 5 point scale from 1 (Not at all or Never) to 5 (Very much or Always)	“In the past 7 days… how run-down did you feel on average?”
Cognitive Function	PROMIS: Item Bank v2.0 – Cognitive Function: 32 items re: cognitive function including frequency and severity of cognitive concerns	Patient rated on a 5 point scale from “very often (several times a day)” (1) to “never” (5)	“In the past 7 days… I have had trouble concentrating”

### Procedure

Once identified, participants will be contacted by telephone or email to further verify inclusion and exclusion criteria, to obtain written informed consent, and schedule their first appointment. We will aim to assess participants at 3 different time points: < 2 months (T1), 3/4 (T2) and 5/6 (T3) months post chemotherapy, with healthy controls ideally being tested on the same day or within 5 days from when the patient is tested.

Participants will then receive a package via mail or in person, with instructions and materials for stool sample collection, and a link to fill out the self-report questionnaires, and demographic and clinical data online using REDCap. REDCap is a free, secure, browser-based application designed to support Electronic Data Capture for research studies provided through the Clinical Research Unit in the Cumming School of Medicine at the University of Calgary. Medical charts will also be accessed to confirm information regarding cancer diagnosis, treatment, glucocorticoid and antibiotic medication use, and mucositis.

Participants will be instructed to collect stool samples at home which can be stored in a freezer up to 3 days prior to their appointment, and to complete their online questionnaires prior to the appointment. On the day of their appointment, we will collect their stool sample from them. Participants will then be taken to have a hair sample collected for cortisol analysis (approximately 10 min), complete the cognitive task (i.e. the SART) on a computer (approximately 6 min), and complete their DXA scan (approximately 10 min), and have their blood drawn for serum biomarker analyses (approximately 10 min). This whole process should take approximately 45 min. Participants’ T2 and T3 month follow-up appointments will be scheduled at each subsequent visit and the procedure will be the same with respect to psychological and cognitive measures completed online, followed by their in person testing and biological sample collection. Participants will be provided a voucher for parking, and may choose from compensation in the form of either $30 at the end of the study or a pass to a fitness class after each appointment (they will have options to choose from in advance). If desired, participants will also receive a package with their individual results summarized and interpreted at the end of the study.

### Data analysis

*Objective 1* will examine whether chemotherapy is related to long-term gut dysbiosis as indicated by reduced alpha diversity in patients relative to controls, and from time points 1 through 3. Gut microbiota alpha diversity will be evaluated with the Shannon and Chao1 indexes using QIIME. To further examine differences in bacterial composition between the groups, LEfSe (linear discriminant analysis effect size) will be used to evaluate differential abundance between patients and controls, allowing us to identify the key bacteria that contribute most to the chemotherapy group having different bacteria relative to healthy controls. Linear Mixed Modelling (LMM) analyses will be used to examine differences across time and between groups. Group (survivors vs. healthy controls) and time (T1 – T3) will be fixed factors and individual subject will be a random factor.

*Objective 2* will first test group differences in gut microbiota, cytokines, LPS, 5-HT and BDNF, cortisol, and body composition using LMM as described above. We will test the interplay between the physiological systems of our model by examining associations between the gut microbiota, cytokines, LPS, 5-HT and BDNF, cortisol, and body composition using univariate correlations. Then multivariate regression models regressing cytokines, cortisol and body composition on gut microbiota diversity will be conducted, including variables that showed significant univariate correlations in the modelling. Covariates included theoretically based on the literature will include type of cancer diagnosis (i.e. blood or solid tumor), history of mucositis, glucocorticoid medication, antibiotic, and probiotic use, birth delivery mode, and breastfeeding in infancy.

*Objective 3* will first test group differences in cognitive and psychological parameters, gut microbiota diversity, and levels of 5-HT and BDNF using LMM as described above. We will test the relationships between gut microbiota, 5-HT and BDNF, and cognitive and psychological parameters using univariate correlations. Then multivariate regression models regressing psychological and cognitive parameters on gut microbiota diversity will be conducted, including variables that showed significant univariate correlations in the modelling. Covariates included theoretically based on the literature will as specified in objective 2.

*Objective 4* will explore relationships between clinical factors and all dependent variables, first using univariate correlations, followed by multivariate regression modelling to examine associations between cancer survivors’ clinical and demographic factors, and the dependent variables of gut microbiota alpha diversity and differential species composition, physiological parameters (i.e. cytokines, LPS, 5-HT, BDNF, and cortisol), body composition, and psychological and cognitive outcomes.

## Discussion

Using the Microbiota-Gut-Brain Axis model and the biopsychosocial framework, this research aims to elucidate the long-term effects of chemotherapy on gut microbiota, and psychological, cognitive, metabolic, and immune outcomes in young adult cancer survivors. Moreover, this study will allow us to systematically explore and validate aspects the novel model proposed here; the Chemotherapy Driven Dysbiosis of the Microbiota-Gut-Brain Axis Model.

The design of this study is not without its limitations and we have taken steps to reduce potential confounds and bias. Although the sample size of this study is relatively small, it is comparable to other studies examining the effects of chemotherapy on gut microbiota, and taking into account feasibility, this sample size will produce adequate statistical power. Furthermore, while most studies typically require participants to have not taken antibiotics for at least 3 months prior to fecal collection, we have set the required time at 1 month. This is to account for the health status of the clinical population, tight timing between follow-up appointments, and to reduce potential attrition. Although colorectal cancers do frequently occur in young adults, we have chosen to exclude this specific diagnosis type from our sample as previous research suggests that specific changes in gut microbiota may be involved in the onset and/or progression of colorectal cancer [56, 57], and therefore present a potential confounding variable. Taking into consideration that the first data collection time point is very close to the final chemotherapy treatment, it is possible that hair sample collection may not be feasible for all patients, in which case hair samples may only be collected at time points 2 and 3 for some patients. However, this should still allow us to examine changes in cortisol over 3-4 and 5- 6 months post-chemotherapy since hair cortisol allows for retrospective cortisol quantification. Finally, as this novel study is exploratory in nature, we think that the methodological plan outlined will provide a rich and varied data set with which we will be able to answer our research questions. This knowledge will ultimately be used to inform future, larger studies and, most importantly, interventions.

## Conclusion

Knowing what bacterial species are depleted after chemotherapy, how long these effects last, and the physiological mechanisms that may drive psychological and cognitive issues among survivors is a crucial step forward in gut microbiota and young adult cancer research. Understanding the bio-behavioural mechanisms that drive psychological and cognitive dysfunction among survivors will allow for tailored interventions to be developed. Future studies can aim to aid cancer patients and survivors by co-administering specific health promoting bacteria (i.e. probiotics), with the potential of preventing or reversing physical and mental health issues that many young survivors face.

## Data Availability

The datasets that will be used and/or analyzed during the current study are not yet available as the study is presently ongoing, but will be available from the corresponding author on reasonable request once recruitment and data collection are complete.

## Abbreviations

5-HT: Serotonin
ACTH: Adrenocorticotropic hormone
ADHD: Attention deficit hyperactivity disorder
ALL: Acute lymphoblastic leukemia
ASD: Autism spectrum disorder
AYA: Adolescent and young adult
BDNF: Brain derived neurotrophic factor
CNS: Central nervous system
CRF: Corticotrophin releasing factor
CRP: C-reactive protein
DSM-IV: Diagnostic and statistical manual of mental disorders 4th edition
DXA: Dual-energy X-ray absorptiometry
ELISA: Enzyme-linked immunosorbent assay
GABA: gamma-Aminobutyric acid
HPA: Hypothalamic-pituitary-adrenal
IL-1ß: Interleukin 1 beta
IL-6: Interleukin 6
INF-α: Interferon alpha
LEfSe: Linear discriminant analysis effect size
LMM: Linear mixed model
LPS: Lipopolysaccharide
NMDS: Non-metric multidimensional scaling
PROMIS: Patient-reported outcomes measurement information system
PTSD: Post traumatic stress disorder
QIIME: Quantitative insights into microbial ecology
SART: Sustained attention to response task
TNF-α: Tumour necrosis factor alpha

## References

[1] Petra AI, Panagiotidou S, Hatziagelaki E, Stewart JM, Conti P, Theoharides TC (2015). Gut-microbiota-brain Axis and its effect on neuropsychiatric disorders with suspected immune Dysregulation. Clin Ther.

[2] Foster JA, Neufeld KM (2013). Gut-brain axis: how the microbiome influences anxiety and depression. Trends Neurosci.

[3] Maes M, Kubera M, Leunis JC, Berk M, Geffard M, Bosmans E (2013). In depression, bacterial translocation may drive inflammatory responses, oxidative and nitrosative stress (O&NS), and autoimmune responses directed against O&NS-damaged neoepitopes. Acta Psychiatr Scand.

[4] Dinan TG, Cryan JF (2017). The microbiome-gut-brain Axis in health and disease. Gastroenterol Clin N Am.

[5] Forsythe P, Bienenstock J, Kunze WA (2014). Vagal pathways for microbiome-brain-gut axis communication. Adv Exp Med Biol.

[6] Bai J, Behera M, Bruner DW (2018). The gut microbiome, symptoms, and targeted interventions in children with cancer: a systematic review. Support Care Cancer.

[7] Jordan KR, Loman BR, Bailey MT, Pyter LM (2018). Gut microbiota-immune-brain interactions in chemotherapy-associated behavioral comorbidities. Cancer.

[8] Jacola LM, Edelstein K, Liu W, Pui CH, Hayashi R, Kadan-Lottick NS, Srivastava D, Henderson T, Leisenring W, Robison LL (2016). Cognitive, behaviour, and academic functioning in adolescent and young adult survivors of childhood acute lymphoblastic leukaemia: a report from the childhood Cancer survivor study. Lancet Psychiatry.

[9] Schulte F, Brinkman TM, Li C, Fay-McClymont T, Srivastava DK, Ness KK, Howell RM, Mueller S, Wells E, Strother D (2018). Social adjustment in adolescent survivors of pediatric central nervous system tumors: a report from the childhood Cancer survivor study. Cancer.

[10] Burkhamer J, Kriebel D, Clapp R (2017). The increasing toll of adolescent cancer incidence in the US. PLoS One.

[11] Borre YE, O'Keeffe GW, Clarke G, Stanton C, Dinan TG, Cryan JF (2014). Microbiota and neurodevelopmental windows: implications for brain disorders. Trends Mol Med.

[12] Cancer CPA (2017). Adolescents and young adults with Cancer: system performance report. In. Toronto, Canada.

[13] Barr RD, Ferrari A, Ries L, Whelan J, Bleyer WA (2016). Cancer in adolescents and young adults: a narrative review of the current status and a view of the future. JAMA Pediatr.

[14] Asher A (2011). Cognitive dysfunction among cancer survivors. Am J Phys Med Rehabil.

[15] Seitz DC, Besier T, Debatin KM, Grabow D, Dieluweit U, Hinz A, Kaatsch P, Goldbeck L (2010). Posttraumatic stress, depression and anxiety among adult long-term survivors of cancer in adolescence. Eur J Cancer.

[16] Grenham S, Clarke G, Cryan JF, Dinan TG (2011). Brain-gut-microbe communication in health and disease. Front Physiol.

[17] Huang Y, Yang W, Liu H, Duan J, Zhang Y, Liu M, Li H, Hou Z, Wu KK (2012). Effect of high-dose methotrexate chemotherapy on intestinal Bifidobacteria, Lactobacillus and Escherichia coli in children with acute lymphoblastic leukemia. Exp Biol Med (Maywood).

[18] Montassier E, Gastinne T, Vangay P, Al-Ghalith GA, Bruley des Varannes S, Massart S, Moreau P, Potel G, de La Cochetiere MF, Batard E (2015). Chemotherapy-driven dysbiosis in the intestinal microbiome. Aliment Pharmacol Ther.

[19] Scully C, Epstein J, Sonis S (2003). Oral mucositis: a challenging complication of radiotherapy, chemotherapy, and radiochemotherapy: part 1, pathogenesis and prophylaxis of mucositis. Head Neck.

[20] Dash S, Clarke G, Berk M, Jacka FN (2015). The gut microbiome and diet in psychiatry: focus on depression. Curr Opin Psychiatry.

[21] Dantzer R, Kelley KW (2007). Twenty years of research on cytokine-induced sickness behavior. Brain Behav Immun.

[22] Berk M, Williams LJ, Jacka FN, O'Neil A, Pasco JA, Moylan S, Allen NB, Stuart AL, Hayley AC, Byrne ML (2013). So depression is an inflammatory disease, but where does the inflammation come from?. BMC Med.

[23] Capuron L, Gumnick JF, Musselman DL, Lawson DH, Reemsnyder A, Nemeroff CB, Miller AH (2002). Neurobehavioral effects of interferon-alpha in cancer patients: phenomenology and paroxetine responsiveness of symptom dimensions. Neuropsychopharmacology.

[24] O'Mahony SM, Hyland NP, Dinan TG, Cryan JF (2011). Maternal separation as a model of brain-gut axis dysfunction. Psychopharmacology.

[25] Beishuizen A, Thijs LG (2003). Endotoxin and the hypothalamo-pituitary-adrenal (HPA) axis. J Endotoxin Res.

[26] Sonis ST (2004). The pathobiology of mucositis. Nat Rev Cancer.

[27] Schurman JV, Singh M, Singh V, Neilan N, Friesen CA (2010). Symptoms and subtypes in pediatric functional dyspepsia: relation to mucosal inflammation and psychological functioning. J Pediatr Gastroenterol Nutr.

[28] Hilimire MR, DeVylder JE, Forestell CA (2015). Fermented foods, neuroticism, and social anxiety: an interaction model. Psychiatry Res.

[29] Steenbergen L, Sellaro R, van Hemert S, Bosch JA, Colzato LS (2015). A randomized controlled trial to test the effect of multispecies probiotics on cognitive reactivity to sad mood. Brain Behav Immun.

[30] Clapp M, Aurora N, Herrera L, Bhatia M, Wilen E, Wakefield S (2017). Gut microbiota's effect on mental health: the gut-brain axis. Clin Pract.

[31] Desbonnet L, Garrett L, Clarke G, Kiely B, Cryan JF, Dinan TG (2010). Effects of the probiotic Bifidobacterium infantis in the maternal separation model of depression. Neuroscience.

[32] Anderson SC, Cryan JF, Dinan T (2017). The Psychobiotic revolution: mood, food, and the new science of the gut-brain connection.

[33] Janssen DG, Caniato RN, Verster JC, Baune BT (2010). A psychoneuroimmunological review on cytokines involved in antidepressant treatment response. Hum Psychopharmacol.

[34] Sarkar A, Harty S, Lehto SM, Moeller AH, Dinan TG, Dunbar RIM, Cryan JF, Burnet PWJ (2018). The microbiome in psychology and cognitive neuroscience. Trends Cogn Sci.

[35] Vazquez E, Barranco A, Ramirez M, Gruart A, Delgado-Garcia JM, Martinez-Lara E, Blanco S, Martin MJ, Castanys E, Buck R (2015). Effects of a human milk oligosaccharide, 2′-fucosyllactose, on hippocampal long-term potentiation and learning capabilities in rodents. J Nutr Biochem.

[36] Prehn-Kristensen A, Zimmermann A, Tittmann L, Lieb W, Schreiber S, Baving L, Fischer A (2018). Reduced microbiome alpha diversity in young patients with ADHD. PLoS One.

[37] Naseribafrouei A, Hestad K, Avershina E, Sekelja M, Linlokken A, Wilson R, Rudi K (2014). Correlation between the human fecal microbiota and depression. Neurogastroenterol Motil.

[38] Prasad PK, Hardy KK, Zhang N, Edelstein K, Srivastava D, Zeltzer L, Stovall M, Seibel NL, Leisenring W, Armstrong GT (2015). Psychosocial and neurocognitive outcomes in adult survivors of adolescent and early young adult Cancer: a report from the childhood Cancer survivor study. J Clin Oncol.

[39] Deleemans JM, Reynolds K, Schulte F (2018). Associations among health behaviours and psychosocial outcomes in adolescent and young adult (AYA) cancer survivors.

[40] Tai E, Buchanan N, Townsend J, Fairley T, Moore A, Richardson LC (2012). Health status of adolescent and young adult cancer survivors. Cancer.

[41] Bervoets L, Van Hoorenbeeck K, Kortleven I, Van Noten C, Hens N, Vael C, Goossens H, Desager KN, Vankerckhoven V (2013). Differences in gut microbiota composition between obese and lean children: a cross-sectional study. Gut Pathog.

[42] Bomhof MR, Paul HA, Geuking MB, Eller LK, Reimer RA (2016). Improvement in adiposity with oligofructose is modified by antibiotics in obese rats. FASEB J.

[43] Dudek-Wicher RK, Junka A, Bartoszewicz M (2018). The influence of antibiotics and dietary components on gut microbiota. Prz Gastroenterol.

[44] Mahana D, Trent CM, Kurtz ZD, Bokulich NA, Battaglia T, Chung J, Muller CL, Li H, Bonneau RA, Blaser MJ (2016). Antibiotic perturbation of the murine gut microbiome enhances the adiposity, insulin resistance, and liver disease associated with high-fat diet. Genome Med.

[45] Turnbaugh PJ (2017). Microbes and diet-induced obesity: fast, cheap, and out of control. Cell Host Microbe.

[46] Effect Size (Cohen's d) Calculator for a Student t-Test [https://www.danielsoper.com/statcalc/calculator.aspx?id=48]. Accessed Feb 2019.

[47] Segata N, Izard J, Waldron L, Gevers D, Miropolsky L, Garrett WS, Huttenhower C (2011). Metagenomic biomarker discovery and explanation. Genome Biol.

[48] Nicolucci AC, Hume MP, Martinez I, Mayengbam S, Walter J, Reimer RA (2017). Prebiotics reduce body fat and Alter intestinal microbiota in children who are overweight or with obesity. Gastroenterology.

[49] Berruti A, Dogliotti L, Terrone C, Cerutti S, Isaia G, Tarabuzzi R, Reimondo G, Mari M, Ardissone P, De Luca S (2002). Changes in bone mineral density, lean body mass and fat content as measured by dual energy x-ray absorptiometry in patients with prostate cancer without apparent bone metastases given androgen deprivation therapy. J Urol.

[50] Sandberg E (2018). Meso scale discovery training report. In: The University of Calgary.

[51] Serotonin ELISA kit [http://www.enzolifesciences.com/ADI-900-175/serotonin-elisa-kit/]. Accessed Feb 2019.

[52] Steudte S, Kirschbaum C, Gao W, Alexander N, Schonfeld S, Hoyer J, Stalder T (2013). Hair cortisol as a biomarker of traumatization in healthy individuals and posttraumatic stress disorder patients. Biol Psychiatry.

[53] Steudte-Schmiedgen S, Kirschbaum C, Alexander N, Stalder T (2016). An integrative model linking traumatization, cortisol dysregulation and posttraumatic stress disorder: insight from recent hair cortisol findings. Neurosci Biobehav Rev.

[54] Cessna JM, Jim HS, Sutton SK, Asvat Y, Small BJ, Salsman JM, Zachariah B, Fishman M, Field T, Fernandez H (2016). Evaluation of the psychometric properties of the PROMIS Cancer fatigue short form with cancer patients. J Psychosom Res.

[55] Allan Cheyne J, Solman GJ, Carriere JS, Smilek D (2009). Anatomy of an error: a bidirectional state model of task engagement/disengagement and attention-related errors. Cognition.

[56] Chen W, Liu F, Ling Z, Tong X, Xiang C (2012). Human intestinal lumen and mucosa-associated microbiota in patients with colorectal cancer. PLoS One.

[57] Sears CL, Garrett WS (2014). Microbes, microbiota, and colon cancer. Cell Host Microbe.

